# Comparative Analysis of Different Isolated Oleaginous Mucoromycota Fungi for Their *γ*-Linolenic Acid and Carotenoid Production

**DOI:** 10.1155/2020/3621543

**Published:** 2020-11-05

**Authors:** Hassan Mohamed, Abdel-Rahim El-Shanawany, Aabid Manzoor Shah, Yusuf Nazir, Tahira Naz, Samee Ullah, Kiren Mustafa, Yuanda Song

**Affiliations:** ^1^Colin Ratledge Center of Microbial Lipids, Shandong University of Technology, School of Agriculture Engineering and Food Science, Zibo 255000, China; ^2^Department of Botany and Microbiology, Faculty of Science, Al-Azhar University, Assiut 71524, Egypt; ^3^University Institute of Diet and Nutritional Sciences, The University of Lahore, 54000 Lahore, Pakistan

## Abstract

*γ*-Linolenic acid (GLA) and carotenoids have attracted much interest due to their nutraceutical and pharmaceutical importance. Mucoromycota, typical oleaginous filamentous fungi, are known for their production of valuable essential fatty acids and carotenoids. In the present study, 81 fungal strains were isolated from different Egyptian localities, out of which 11 Mucoromycota were selected for further GLA and carotenoid investigation. Comparative analysis of total lipids by GC of selected isolates showed that GLA content was the highest in *Rhizomucor pusillus* AUMC 11616.A, *Mucor circinelloides* AUMC 6696.A, and *M. hiemalis* AUMC 6031 that represented 0.213, 0.211, and 0.20% of CDW, respectively. Carotenoid analysis of selected isolates by spectrophotometer demonstrated that the highest yield of total carotenoids (640 *μ*g/g) was exhibited by *M. hiemalis* AUMC 6031 and *M. hiemalis* AUMC 6695, and these isolates were found to have a similar carotenoid profile with, *β*-carotene (65%), zeaxanthin (34%), astaxanthin, and canthaxanthin (5%) of total carotenoids. The total fatty acids of all tested isolates showed moderate antimicrobial activity against *Staphylococcus aureus* and *Salmonella Typhi*, and *Penicillium chrysogenum*. To the best of our knowledge, this is the first report on the highest yield of total lipid accumulation (51.74% CDW) by a new oleaginous fungal isolate *R. pusillus* AUMC 11616.A. A new scope for a further study on this strain will be established to optimize and improve its total lipids with high GLA production. So, *R. pusillus* AUMC 11616.A might be a potential candidate for industrial application.

## 1. Introduction

Fatty acids are essential components of membrane lipids and for human nutrition. Polyunsaturated fatty acids (PUFAs) have many health benefits for the human body, particularly in preventing cardiac diseases, maintaining sturdy skeletal muscle, and modulating the nervous and immune systems [1, 2]. PUFAs are traditionally extracted from animals and plants but can be alternatively derived from oleaginous microbes. Among microbes, oleaginous fungi are suitable sources of PUFAs. PUFA production from fungi is sustainable and independent of climatic or seasonal changes. Oleaginous fungi are able to grow on a wide variety of substrates with relatively high growth rates, while the oils produced are of high purity [3]. In recent years, the demand for alternative and sustainable sources of PUFAs has increased. PUFAs such as omega-3 and omega-6 compounds produced by microbial origin have attracted much attention. *γ*-Linolenic acid (GLA) (6,9,12,*cis*,*cis*,*cis*-octadecatrienoic acid) is an omega-6 PUFA, the precursor of prostaglandin. GLA plays an important role in human health, especially improving health in patients who are suffering from diabetes, aging, cancer, and immune diseases [4–6].

The most reliable alternative way to produce high value-added lipids (i.e., single cell oils) is probably through oleaginous fungi. Among fungi, order *Mucorales* are considered potential candidates for application in industrial PUFA production [7]. A large number of Zygomycota fungi have been reported as oleaginous [8, 9]. Ratledge performed extensive screening of more than 300 Mucoromycota fungi (13 genera) based on several criteria to find the best GLA producer [10]. In this connection, *M. circinelloides* strain WJ11 can produce high lipid content up to 36% (*w*/*w*) of cell dry weight (CDW), which is 2.3-fold more than in *M. circinelloides* strain CBS 277.49 which produce 15% lipid of CDW demonstrated by Tang et al. [11]. On the other hand, *M. isabellina* ATHUM 2935 produced maximum lipids of 8.5 g/L, 83.3% (*w*/*w*) in dry cell weight (DCW), and conversion yield per unit of glucose consumed (0.25 g/g) [12].

Besides essential fatty acid values, carotenoids are one of the most important groups of natural fat-soluble pigments widely represented in nature with diverse functions and they have a valuable biotechnological interest [13, 14]. Carotenoids are widely used in the food, pharmaceutical, and cosmetic industries and as food color additives. Recently, carotenoids attracted great attention, due to their various beneficial effects on human and animal health; for example, their antioxidant property linked with a preventive action on different types of cancer [15] and immune system enhancement [16] provides color to egg yolks in poultry and to the flesh of some fish or crustaceans' shells in aquaculture. Most of the carotenoids are produced by chemical synthesis, and only a few natural compounds can be obtained from cheap plant sources [17], and some fungi are used as industrial carotenoid producers [18]. *β*-Carotene, one of the most ubiquitous carotenes in nature, it is usually found in Mucorales fungi, as shown in *Phycomyces blakesleeanus* [18], *M. circinelloides*, and *Blakeslea trispora* [19]. Thus, fatty acids and carotenoids derived from oleaginous microbes could be the basis for the discovery of new therapeutic agents and searching for novel fungal strains is considered an urgent task.

The aim of the present study was to search the potent strains of Mucoromycota spp. that has the potential to produce high *γ*-linolenic acid and carotenoids. In this study, several filamentous Mucoromycota spp. were isolated from different ecoregions of Egypt and were investigated for their cell mass, total lipid content, GLA, and carotenoid production.

## 2. Materials and Methods

### 2.1. Sample Collection and Isolation of Fungi

Samples from different sources were collected and transferred into sterilized polyethylene bags; each bag was labeled appropriately by indicating the site of collection, time, date, and place of collection. Finally, the samples were then taken to the laboratory under aseptic conditions. The isolation of filamentous fungi from soils, animal dungs, and textile was conducted by the serial decimal dilutions on the potato dextrose agar (PDA) medium containing potato infusion (200 g/L), dextrose (20 g/L), and agar (20 g/L) into 1000 mL distilled H_2_O with final pH (5.6 ± 0.2) while the sources of vegetables and laryngitis case were performed by a direct plate method and plates amended with an antibacterial agent, e.g., chloramphenicol (0.2 g/L). All plates were then incubated at 28°C for 4–7 days. Every single fungal colony was purified and morphologically identified, then preserved on slants containing the same culture medium and stored at 4°C, for further investigations. Light microscopic examination of the isolated fungi was done to check the purity and structure of the isolates.

### 2.2. Molecular Identification of Fungal Cultures

#### 2.2.1. DNA Extraction

Isolated fungal strains were grown on PDA plates for 3–4 days, and fungal cell mass was collected and grinded as powder by using liquid nitrogen. Genomic DNA from powered fungus was extracted by the DNA quick Plant System isolation kit (Tiangen, Biotech, Beijing, Co., Ltd., China), according to the manufacturer's instructions. After elution, DNA was preserved at −20°C until future analyses.

#### 2.2.2. PCR Amplification

The 18S rRNA encoding gene was amplified by the polymerase chain reaction (PCR) from purified genomic DNA using fungal-specific primers: ITS1 (TCCGTAGGTGAACCTGCGG) and ITS4 (TCCTCCGCTTATTGATATGC) (Sangon Biotech). The PCR amplification was performed by using the 2X Accurate *Taq* Master Mix Kit (Qiagen). Each PCR reaction mixture was combined in a total volume of 25 *μ*L including about 50 ng of template DNA, 12.5 *μ*L PCR Master Mix, and 0.5 *μ*L each of ITS1 and ITS4, and the total reaction volume was completed by 11.5 *μ*L of DNase-free water. The complete reaction of the mixture was then applied to the thermal cycle (Master cycler, Eppendorf, Germany) under the following programmed PCR conditions: temperature cycling comprised 35 cycles of DNA denaturation at 95°C for 10 min, followed by annealing at 56°C for 30 seconds and extension at 72°C for 1.45 min, and final extension for 3.30 min at 72°C to complete the synthesis of all strands. The PCR products were analyzed by electrophoresis on 1% (*v*/*v*) agarose TBE-gels (Tris-base Boric EDTA-gel), and the gels were visualized via a gel documentation system (Syngene, USA); then, the PCR product was purified from the agarose gel utilizing QIA quick DNA gel extraction kit (Omega Bio-tek) according to the manufacturer's instructions.

#### 2.2.3. DNA Sequencing and Phylogenetic Analysis

Purified DNA amplicons were sent to Sangon Biotech (Shanghai) Co., Ltd. for sequencing. The DNA sequences from all fungal isolates were aligned with the closely related species sequences obtained from the NCBI nucleotide database using BLAST (https://blast.ncbi.nlm.nih.gov/Blast.cgi). The phylogenetic analyses inferred from the data were constructed using MEGA-X software version 10.1.8. [20]. Alignments were performed with Clustal Omega [21]. The phylogenetic reconstruction was performed by the neighbor-joining (NJ) method with an alignment of the prepared sequences; the stability of phylogenetic tree branches was estimated with a *p*-distance substitution model and bootstrapping of 1000 replications [22].

### 2.3. Lipid Fermentation and Cultivation Conditions

The lipid fermentation medium used during all screening stages of the *Mucorales* strains was rich in carbon source and limited in nitrogen source to induce lipid accumulation. For each strain, 100 *μ*L of spore suspension approximately 10^6^~107 spores/mL was initially inoculated into 150 mL of Kendrick and Ratledge (K and R) medium [4] in 1 L baffled flasks after medium sterilization at 121°C for 20 min, according to Hussain et al. [23]; this medium contained glucose (30.0 g/L), ammonium tartrate (3.30 g/L), KH_2_PO_4_ (7.0 g/L), Na_2_HPO_4_ (2.0 g/L), MgSO_4_·7H_2_O (1.50 g/L), yeast extract (1.50 g/L), CaCl_2_·2H_2_O (0.1 g/L), FeCl_3_·6H_2_O (8.0 mg/L), ZnSO_4_·7H_2_O (1.0 mg/L), CuSO_4_·5H_2_O (0.1 mg/L), Co(NO_3_)_2_·6H_2_O (0.1 mg/L), and LMnSO_4_·5H_2_O (0.1 mg/L). All components were completely dissolved in 1 L H_2_O. The cultures were incubated for 24 h at 30°C with shaking at 150 rpm (Chengdu YIKE Instrument Co., Ltd., China, ZWYR-C211D) and then employed at 10% (*v*/*v*) to inoculate 1 L baffled flasks containing 150 mL modified K and R (Nitrogen limiting) medium the same mentioned composition except glucose 80.0 g/L and ammonium tartrate 2.0 g/L. The cultures were incubated for 4 days at 30°C with shaking at 150 rpm.

### 2.4. Carotenoid Production

For screening of carotenoids, the selected strains were cultured (the same spore suspension mentioned above) separately on 150 mL YPG medium containing yeast extract (3 g/L), peptone (10 g/L), glucose (20 g/L), distilled water (1 L), and pH at 4.5 ± 0.2 in 1 L baffled flasks. The cultures were incubated for 24 h at 28°C with shaking at 150 rpm; then 10% (*v*/*v*) were transferred to inoculate 1 L baffled flasks containing the same medium, grown for 4 days under continuous shaking with light.

### 2.5. Determination of Cell Dry Weight (CDW)

The fungal cell mass was harvested by filtration according to Hussain et al. [23]. Briefly, a suction filtration method by Buchner funnel was employed followed by 3 washes with distilled water to eliminate possible medium contents. After filtration, all samples were then frozen overnight at – 80°C, and the samples were lyophilized (freeze-dried) for 48 h, and the cell dry weight was measured by the weighing method.

### 2.6. Total Lipids and Fatty Acid Profiling

After biomass collection by filtration, the lipid extraction was performed with minor modifications according to the Folch method as described previously by Khan et al. [24]. Briefly, frozen ~20 mg of biomass was vigorously homogenized with chloroform/methanol (2 : 1 *v*/*v*). Methanolic HCl when 10% (*v*/*v*) was used for methylation at 60°C for 4 h pentadecanoic acid (15 : 0 from Millipore, Sigma-Aldrich, USA) was added into the freeze-dried cell as an internal standard before methylation. Finally, the fatty acid methyl esters (FAMEs) were extracted with *n*-hexane and subsequently analyzed by gas chromatography (GC) by using the DB-Waxetr column with the following specifications: film thickness (0.25 *μ*m), 30 mm × 0.32 mm (Shimadzu Co., Ltd., Japan). The GC operating conditions were as follows: 120°C for 3 min, ramp to 200°C at 5°C min, continuous ramping of temperature to 220°C at 4°C min, and then held at 220°C for 2 min [24]. Obtained chromatographic peaks and their retention times were determined by the comparison to fatty acid methyl ester (FAME) standard mixture (Supelco 37-Component FAME Mix, Sigma-Aldrich, MO, USA).

### 2.7. Carotenoid Extraction and Analysis

Carotenoid extraction was done as described previously by Naz et al. [25]. Briefly, 100 mg of mycelia powder was taken and dissolved in 1 mL hexane in a 10 mL screw tube, and vortexing was done. This extraction step was repeated until the pellet was found to be devoid of pigments. Extracts were combined and then partitioned with an equal volume of 10% diethyl ether in petroleum ether. To facilitate the separation and to remove dissolved hexane, 2 mL distilled water was added. The petroleum ether fractions were combined and dried under nitrogen gas [26], until spectrophotometric analysis. Measurements of carotenoid content in the dry biomass (%) including *β*-carotene, astaxanthin, zeaxanthin, and canthaxanthin were determined spectrophotometrically by using their standard method according to Li et al. [27]. Standards were purchased from Sigma-Aldrich (St. Louis, USA). For carotenoid quantification by a spectrophotometer, stock solutions of external standards were prepared by dissolving 1 mg of standard in 1 mL of tetrahydrofuran (THF). Calibration curves were made with following concentrations: 2, 5, 10, 15, 20, 25, 30, 40, 50, 60, 80, and 100 *μ*g/mL. For sample analysis, dried extracts were resuspended in 700 *μ*L of THF and 200 *μ*L of each sample was taken into 96-well microplate and then the concentration was determined using an absorbance plate reader (UV/Vis Multiskan Sky Microplate Spectrophotometer, Thermo Fisher Scientific™), by their spectral data by using individual standard curves. The absorbance of samples was taken at a wavelength of 530 nm for astaxanthin and 450 nm for *β*-carotene, zeaxanthin, and canthaxanthin. Carotenoid content was calculated based on the calibration curve equation in which *y* was the absorbance value measured and *x* was the carotenoid concentrations (*μ*g/g).

### 2.8. Testing the Biological Activity of Extracted Lipids

#### 2.8.1. Microorganisms

The antimicrobial potential of the total lipids extracted from selected eleven fungal strains and lipids was evaluated against human pathogenic microorganisms as referenced cultures obtained from the American Type Culture Collection (ATCC) included Gram-positive bacteria: *Bacillus subtilis* ATCC 6633 and *Staphylococcus aureus* ATCC 6538P, and Gram-negative bacteria: *Escherichia coli* ATCC 8739 and *Salmonella typhimurium* ATCC 14028, as well as toxigenic fungi: *Aspergillus flavus* ATCC 16883 and *Penicillium chrysogenum* ATCC 18226. The bacterial species were grown at 37°C for 24 h and maintained on a NA nutrient agar medium (5 g/L peptone, 3 g/L beef extract, 5 g/L sodium chloride, 15 g/L agar, 1 L distilled water, and 7.4 ± 0.2 pH at 25°C), and fungal species were grown at 28°C for 5 days and kept at PDA medium potato dextrose agar (200 g/L potato infusion, 20 g/L dextrose, 15 g/L agar, 1 L distilled water, and 5.8 ± 0.2 pH at 25°C); then all strains were stored at 4°C for a further study.

#### 2.8.2. Antimicrobial Evaluation

Antimicrobial activities of extracted total lipids were evaluated using a well diffusion method as described by Fotoon et al. [28] on Mueller-Hinton agar (MHA) and (PDA). From extracted lipids as described above, 0.1 g of total lipids was dissolved in 0.9 mL of 10% DMSO, sterilized by filtration using a sintered glass filter, and stored at 4°C. The appropriate solidified medium MHA and PDA plates were inoculated with bacterial and fungal strain separately under aseptic conditions to a concentration of 1.5 × 10^5^ CFU/mL, and wells (diameter~6 mm) were filled with 50 *μ*L of the test lipid stock; plates were left on the fridge till compound saturation into wells and then incubated at 37°C for 24 h and 28°C for 3–5 days for bacteria and fungi, respectively. After the incubation period, the diameter of the growth inhibition zones was measured in millimeter (mm). DMSO (10%) was used as a negative control while chloramphenicol (0.01 g/mL) and fluconazole (final solution of 0.06 g/mL) were used as a positive control. All tests were performed in triplicate.

#### 2.8.3. Minimal Inhibitory Concentration (MIC) Assay

MICs of extracted lipids were analyzed quantitatively against human pathogens. The fresh bacterial and fungal cell suspension was diluted in tubes containing 10 mL of MHB and YPG broth and was adjusted to get final inoculum to 6 × 10^5^ CFU/mL (a positive control tube only has medium). The antimicrobial activity of lipids was prepared to get dilution series (25, 50, 75, and 100 mg/mL); then the mixture was mixed. The tubes were incubated at 37°C from 18 to 24 hours for bacteria and 28°C from 3 to 4 days in case of fungi before the recording of results. The lowest concentration of lipids that completely inhibits the growth of the bacteria and fungi in the tubes (no turbidity) was determined as the MIC for each pathogen [29].

### 2.9. Data Analysis

All the experiments were conducted in triplicate, and one-way analysis of the variance was performed using SPSS package followed by Tukey's multiple comparison test. Results were presented as the mean ± SD. All the calculations were done by using GraphPad prism 6 (GraphPad Software, San Diego, CA, USA, https://www.graphpad.com). Differences were considered statistically significant for *p* < 0.05.

## 3. Results

### 3.1. Morphological Characterization of Fungal Strain

Fungi are valuable resources for lipid production containing high fractions of polyunsaturated fatty acids and have the potential to serve as a source of significant quantities of important bioactive substances. In this study, 81 different strains (Supplementary material Table S1) were obtained from varied ecological sources in Egypt, and among them, 11 isolates mainly from Mucoraceae (Table 1) were selected and investigated according to their importance in lipid and fatty acid production. A variety of macroscopic structures were observed during their cultivation of fermentation medium under the lipid accumulation and fermentation process. Eleven strains, mainly from *Mucor* spp., grew in a scattered hyphal form, and only two strains, coded as AUMC 11641 and AUMC 6697.A, grew in the form of pellets during fermentation with different sizes, while the other remaining strains showed mixed macroscopic morphology; the viscous growth was observed for these strains because dispersed mycelium and fluffy filamentous were more prone to attach to the wall, which resulted in more pronounced sporulation.

**Figure 1:**
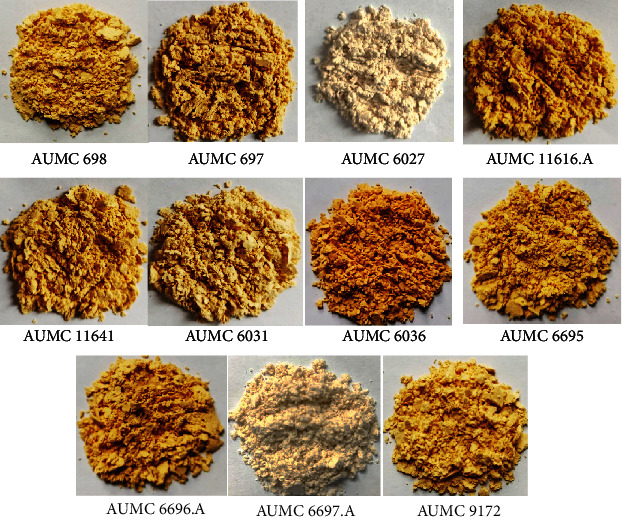
Lyophilized biomass of isolated *Mucor* strains grown on lipid accumulation media.

We found differences in the color of lyophilized fungal biomass of selected strains. Two strains, AUMC 6027 and AUMC 6697.A, showed white color while other strains showed normal yellow to intense yellowish orange; this may be due to the production of carotenoids and other pigments (Figure 1).

### 3.2. Phylogenetic Analysis of Oleaginous Fungal Strains

**Figure 2:**
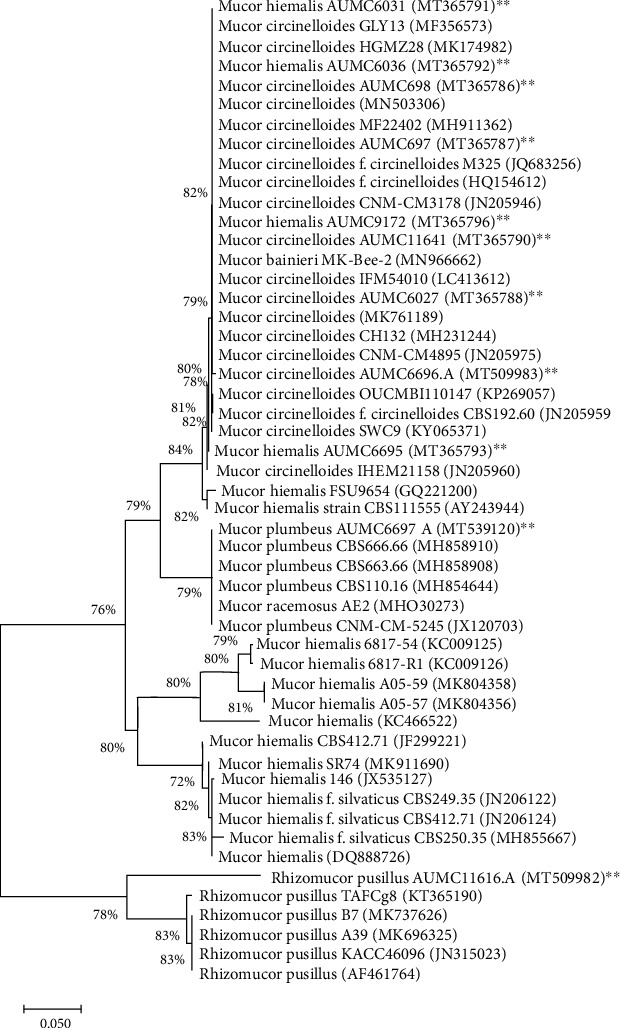
Neighbor-joining (NJ) phylogenetic tree based on ITS-rDNA sequences of tested fungi aligned with closely related strains accessed from the GenBank. Bootstrap values included 1000 replicates for the neighbor-joining method using software MEGA-X (Molecular Evolutionary Genetics Analysis; version 10.1.8). ∗∗ indicate tested strains.

The selected strains were further identified by a rDNA gene sequence method. Based on the ITS region sequencing results, a phylogenetic tree was constructed to describe the similarity of fungal isolates, all 11 oleaginous fungal strains belong to Mucorales. All the sequences were deposited in NCBI data GenBank given with accession number (Figure 2). The sequences were clustered into the Mucorales group, and phylogenetic analysis indicated that the nucleotide sequence of 4 isolates: AUMC 698, AUMC 697, AUMC 6027, and AUMC 11641, reached up to 100% closely matched with *M. circinelloides*, while AUMC 6696.A isolate reached up to 99% closely similar to *M. circinelloides.* Also, the nucleotide sequence of 4 strains: AUMC 6031, AUMC 6036, AUMC 6695, and AUMC 9172, was 97-98% similar to *M. hiemalis*, whereas AUMC 11616.A showed 91% sequence similarity with *Rhizomucor pusillus* and AUMC 6697.A had 100% closely matching with *M. plumbeus*.

### 3.3. Biomass Concentration and Total Lipid Yields

Total lipids are the generic names assigned to a group of fat soluble compounds (such as triacylglycerols, phospholipids, and sphingolipids) found in the organisms. All the selected fungal isolates were grown in the fermentation media for 4 days for their total lipid content. Fungal biomass concentration and total lipid content of investigated *Mucorales* cultured on modified K and R media are reported in Table 2; all eleven strains showed abundant biomass. The fungal biomass of all selected isolates was lyophilized by a freeze drier for 2 days. The quantity of dry cell mass obtained was in the range of 6.38 to 10.44 g/L, of which AUMC 6697.A showed the highest biomass (10.44 g/L) and AUMC 6027 showed typically lower biomass (6.38 g/L). All studied fungal strains of *Mucor* spp. have shown to produce varied total lipid contents ranging from 21.74 to 51.74% (*w*/*w*) of dry biomass (Table 2). The highest lipid content among all tested fungi was found in two strains AUMC 11616.A and AUMC 6696.A with 51.74 and 43.71%, respectively (4.0 g/L). The lipid content of AUMC 6027 was a moderate with the value of 35.26%, and AUMC 6697.A was the lowest lipid content producer strain with the value of 21.74%.

### 3.4. Fatty Acid Profiles of Fungal Strains

**Figure 3:**
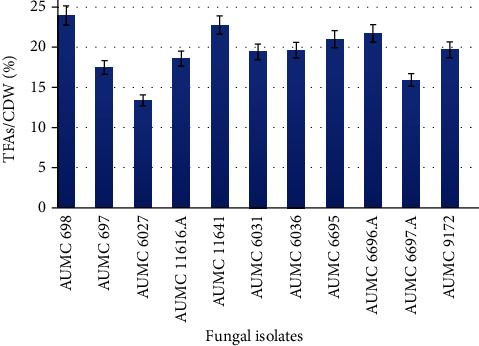
Total fatty acid (TFA) production in Mucor-tested strains. Error bars represent standard deviations (*n* = 3).

Fatty acids are a long chain of hydrocarbons with a carboxylic acid group at one end, and they can be either saturated or unsaturated. Fatty acid analysis is the method by which the composition and concentration of free fatty acids (FFA) in different samples can be analyzed. In this experiment, the free fatty acid composition for all oleaginous strains was analyzed by the GC method. All the fungal strains in the present study were found to contain a high fraction of saturated and monounsaturated fatty acids, mainly myristic acid (C16) and oleic acid (C18) series on each fungus, which is considered a potential feature to indicate the oil quality of fungal based lipids. The main lipids were myristic acid (C14:0), palmitic acid (C16:0), palmitoleic acid (C16:1), stearic acid (C18:0), oleic acid (C18:1), linoleic acid (C18:2), and *γ*-linolenic acid (C18:3). Total fatty acid production of the oleaginous fungi was found to be in the range 13.39 to 23.9% (*w*/*w*) of CDW. The contents of myristic acid (C14:0), palmitoleic acid (C16:1), and linoleic acid (C18:2) reached a maximum of 15.93, 12.43, and 14.88%, respectively, by the fungus AUMC 6027; however, these strains showed the minimum amount of other tested FAs. Notably, the highest production of C16:0, C18:0, and C18:1 was achieved by three strains AUMC 11641, AUMC 6695, and AUMC 6697.A with values 25.16, 6.31, and 44.48%, respectively. Meanwhile, for other remaining fungal strains resulting in fatty acid methyl esters, their profiles were very close to each other. Detailed fatty acid profile of all tested *Mucorales* fungi is shown in Table 3 and Figure 3.

**Figure 4:**
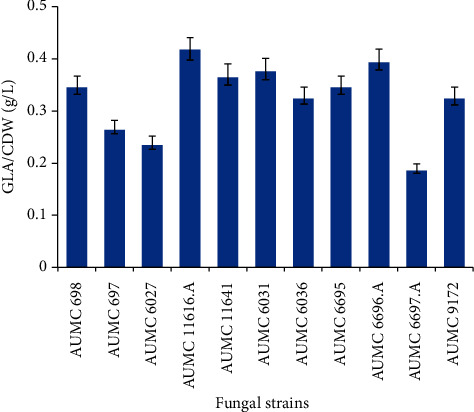
GLA content (g/L) of CDW in *Mucor*-screened strains. Error bars represent standard deviations (*n* = 3).

From the fatty acid analysis confirmed by GC, it was found that selected isolates produce a varied amount of GLA ranging from 7.87 to 10.65%. Our results confirmed that the tested strains were relatively efficient in the production of *γ*-linolenic acid (GLA) in normal shake flask conditions. Based on our results, AUMC 11616.A, AUMC 6696.A, and AUMC 6031 produced the maximum amount of GLA of 0.42, 0.40, and 0.38 g/L, respectively, where seven strains, AUMC 11641, AUMC 689, AUMC 6695, AUMC 6036, AUMC 9172, AUMC 697, and AUMC 6027, showed that the second maximum GLA content in the oil from all tested strains varied from 0.37 to 0.24 g/L, as shown in Figure 4, and the minimum content of GLA in fungal oil was achieved in AUMC 6697, with a level of 0.19 g/L.

### 3.5. Screening of 11 Fungal Strains for Their Production of Carotenoids

**Figure 5:**
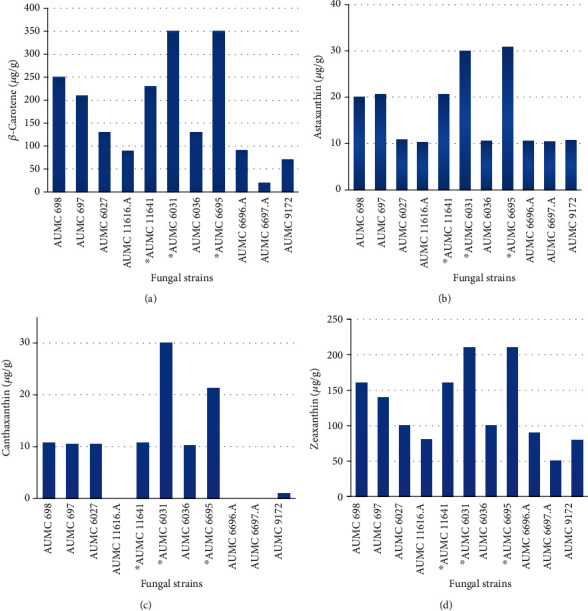
Investigated 11 fungal strains and their overall total carotenoids, (a) *β*-carotene, (b) astaxanthin, (c) canthaxanthin, and (d) zeaxanthin. ∗ indicates highly pigment producer isolates.

The major carotenoid concentration evaluated in the tested fungal strains was the *β*-carotene, astaxanthin, zeaxanthin, and canthaxanthin, after 4 days of fermentation on YPG medium under continuous light. The results found that *β*-carotene and zeaxanthin were produced in a higher amount as compared to canthaxanthin and astaxanthin in *M. hiemalis* while in the case of *M. circinelloides* the major carotenoids were also *β*-carotene and zeaxanthin but astaxanthin and canthaxanthin were produced in a lower amount. In the current study, it was observed that only 2 strains, i.e., *M. hiemalis* AUMC 6031 and AUMC 6695, produced substantial levels of all 4 screened carotenoids as compared to other *Mucor* strains. These two strains produced comparable total carotenoid, i.e., 640 *μ*g/g, but in terms of carotenoid content, slight differences were observed for all screened carotenoid. Carotenoid profiles of all 11 fungal strains are shown in Figure 5.

Among 11 fungal strains whose pigment levels were screened, AUMC 6695 produced higher amount of screened carotenoid as compared to other isolates, i.e., 355 *μ*g/g of *β*-carotene and 215 *μ*g/g of zeaxanthin, while in case of canthaxanthin and astaxanthin, they are recorded as 23 to 38 *μ*g/g, respectively, (Figure 5). In all *Mucorales* strains, *β*-carotene was found to be a dominant carotenoid as compared to other carotene contents. It was also observed that all tested fungal strains produced high quantity of *β*-carotene and zeaxanthin as compared to other carotenoids. Among tested strains, AUMC 698, AUMC 697, AUMC 6027, and AUMC 11616.A showed produced *β*-carotene of 25.0, 20.19, 13.71, and 9 *μ*g/g, respectively, and 16.5, 14.17, 10.26, of 8.1 *μ*g/g of zeaxanthin, respectively. Isolate AUMC 6697.A was found to be the lowest producer of *β*-carotene and zeaxanthin of 2.5 and 5.4 *μ*g/mL, respectively, as confirmed by its mycelial appearance (white color). Furthermore, it is well known that the acetyl-CoA pool is shared by both FAS and carotenoid pathways, so in this strain, more acetyl CoA is being used by the FAS pathway rather than the carotenoid pathway. From the data, it was concluded that *M. hiemalis* strains were significant to produce a high amount of carotenoids rather than *M. circinelloides* strains.

Moreover, all fungal strains were also found to produce astaxanthin and canthaxanthin in a lower amount. AUMC 698, AUMC 697, AUMC 6027, and AUMC 11641 showed moderate concentration of astaxanthin (2.1, 2.7, 1.9, and 2.6 *μ*g/g) and canthaxanthin (0.8, 1.5, 0.5, and 1.8 *μ*g/g, respectively). Interestingly, AUMC 6036 accumulated the lowest range of astaxanthin and canthaxanthin (1.6 to 0.2 *μ*g/g, respectively). Other strains, such as AUMC 11616.A, AUMC 6696.A, AUMC 6697.A, and AUMC 9172, were not efficient in the production of canthaxanthin, but they produced a relatively moderate concentration of astaxanthin (1.3, 1.6, 1.5, and 1.7 *μ*g/g, respectively).

### 3.6. Biological Activity of Total Lipids

Four pathogenic bacteria (*B. subtilis*, *S. aureus*, *E. coli*, and *S. typhimurium*) and two toxogenic fungi (*A. flavus* and *P. chrysogenum*) were selected for the antimicrobial activities of extracted lipids. Compared to the positive control, all the eleven fungal extracts have resulted in some antibacterial and antifungal activity against all the tested pathogens. As shown in Table 4, the well diffusion analysis results (50 *μ*L/well; 10% Conc. dissolved in diluted DMSO) revealed that the antimicrobial inhibition zones were ranged from 11 to 17 mm.

Lipid extracts of AUMC 6036 showed the highest antibacterial effect against *S. aureus* with an inhibition zone of 16.0 mm, followed by AUMC 698, AUMC 11641, and AUMC 6031 with values of 15 mm, while lipid extract of AUMC 6697.A and AUMC 11641 had the maximum antibacterial activity against *E. coli* and *S. typhimurium* with an inhibition zone value of 15 mm. All lipid extracts showed moderate activity against *B. subtilis* with a zone of inhibition range of 12-14 mm. As similar, other remaining tested fungal lipids against tested bacteria. Further, the bioactivity of extracted lipid from AUMC 11641 showed significant antifungal activity against *P. chrysogenum* with a 17.0 mm inhibition zone followed by AUMC 11616.A, AUMC 6031, and AUMC 6696.A with a 16.0 mm inhibition zone. Also, AUMC 697, AUMC 11641, and AUMC 6695 also illustrated the highest antifungal activity against *A. flavus.* All remaining fungal lipids showed moderate antifungal activity against both toxigenic fungi ranging from 12 to 15 mm.

### 3.7. Minimum Inhibitory Concentration (MIC)

Among the tested human pathogens, MIC for the Gram-positive and Gram-negative bacteria tested was determined at the concentration of 50-100 mg/mL, whereas *S. aureus* was the most resistant bacterium to fungal lipids of AUMC 698, AUMC 11641, AUMC 6031, and AUMC 6036, followed by *S. typhi* against AUMC 11641 with a MIC value of 100 mg/mL. Also, *B. subtilis* and *E. coli* were the moderate susceptible species to all studied fungal lipids with MIC values of 50-75 mg/mL. MIC of the fungi was determined as high as 25-100 mg/mL (Table 5). *P. chrysogenum* was the most susceptible fungi against AUMC 11616.A, AUMC 11641, AUMC 6031, AUMC 6696.A, and AUMC 6697.A with MIC values of 25 mg/mL. On the other hand, *A. flavus* was the moderate susceptible fungus to all studied fungal lipids with MIC values of 50-75 mg/mL, though all the tested organisms were inhibited by all tested fungal strains.

## 4. Discussion

Filamentous fungi and yeasts have been considered favorable oleaginous microorganisms [30]. Among the oleaginous fungi, several species of Zygomycetes are very important, characterized by their capability of accumulating high amounts of lipid in their cell biomass. Fungal lipid is stored as droplets within the cells mainly in the form of triacylglycerol and has been considered a valuable and renewable alternative source for the production of high-value products including the polyunsaturated fatty acids (PUFAs) such as *γ*-linolenic acid (GLA). Therefore, the samples were collected from the different ecoregions of Egypt for the isolation of oleaginous fungi. Egyptian ecosystems are a promising source for oleaginous fungi with diverse beneficial bioproduct, presupposing that it may be necessary to explore and characterize such as these fungi for the production of high quantities from PUFAs, pigments, and other related metabolites, and all selected fungal strains were confirmed as *Mucorales.* Isolation and development of fungal strain that can utilize a wide range of low-cost substrates are highly anticipated in the industry for commercial-scale production.

All selected fungi had a varied total lipid content ranging from 21.47 to 51.74% (*w*/*w*) of dry biomass with one strain; AUMC 11616.A produced approximately (51.74%) of total lipid content, comparable to that of the high lipid (36%), producing strain of *M. circinelloides* WJ11 [11]. These results confirmed that our selected filamentous fungi were similar to other commonly used oleaginous microalgae and yeast species [31]. Thus, numerous efforts have been conducted to develop sustainable and applicable techniques for the enhanced production of lipids from these microorganisms [32]. In addition to the findings previously published by Yubin et al. [33], it was shown that the highest biomass and lipid production using glucose as carbon source were achieved by *Mucor circinelloides*, *M. plumbeus*, and *Cunninghamella elegans* by reaching the dry cell weight (CDW) of 4.0, 4.8, and 6.4 g/L and total lipids of 23.8, 20.6, and 33.6%, respectively. On the other hand, *M. rouxii* showed a significant difference in biomass and lipid contents (4 g/L and 13%, respectively), reported by Sukanya et al. [34]. In addition, *M. isabellina* ATHUM 2935 produces maximum lipid at 83.3% (*w*/*w*) whereas 0.25 glucose was consumed per unit under shake-flask fermentation [12]. Moreover, in many previous studies focusing on lipid content in *Mucorales* spp., it was stated that there were 12% in *M. hiemalis* FRR 5101 and 32% in *M. hiemalis* UBOCC-A-101359 lipid content in their dry biomass [35]. Our results demonstrated that biomass and lipid productivity efficiency of the selected *Rhizomucor pusillus* AUMC 11616.A is much higher than that of published before without genetic modification making that as a potential candidate to be developed for industrial application.

The FA compositions, which were analyzed by GC showed that the lipid is mainly composed of myristic acid (C14:0), palmitic acid (C16: 0), palmitoleic acid (C16:1), stearic acid (C18:0), oleic acid (C18:1), linoleic acid (C18:2), and *γ*-linolenic acid (C18:3), in agreement with the FA composition of other *Mucor spp*. [36]. Similar to our findings, oleic acid was the most predominant FA concentration in *M. isabellina* ATHUM 2935 which is more than 50% (*w*/*w*) of the total lipids [37]. Also, fermentation of *Cunninghamella echinulata* on glucose can produce palmitic acid, stearic acid, oleic acid, linoleic acid, and *γ*-linolenic acid at a concentration of 17.2, 6.3, 49.4, 15.8, and 10.4%, respectively [12].

The GLA (*γ*-linolenic acid) is of the commercial interest due to its significant nutritional value and medicinal benefits in treating and preventing several diseases including diabetes, cardiovascular, cancers, and inflammatory disorders [38, 39]. This is the first report for GLA production by selected *R. pusillus* AUMC 11616.A (0.42 g/L), based on lipid (51.73%); if compared with other Mucorales fungi, we found that *M. mucedo* CCF-1384 (0.38 g/L) [40], *M. circinelloides* CBS 203.28 (0.51 g/L) [41], *M. circinelloides* CBS 172-27 (0.22 g/L) [42], *Mucor* sp. LGAM 366 (0.18 g/L) [43], *Mortierella ramanniana* CBS 478.63 (0.44 g/L) [44], *Mucor rouxii* CBS 416.77 (0.32 g/L) [45], *C. echinulata* CCF-103 (0.37 g/L) [40], and C. sp. LGAM (9)2 (0.26 g/L) [46], when cultivated on high-glucose content media.

Carotenoids represent one of the largest groups of natural antioxidants (more than 600 are structurally characterized) and have significant biological effects and various applications in industries [47]. Over the past 2 decades, many studies have been done on numerous oleaginous fungi that are a natural source of carotenoids such as *Neurospora crassa*, *Blakeslea trispora*, *M. hiemalis*, *M. circinelloides*, and *Phycomyces blakesleeanus* [48]. From the literature survey, it was observed that most of the strains of Zygomycetes have dual property of producing GLA, as well as carotenoids [49]. In our study, the highest carotenoid content was observed in the strain AUMC 6695, i.e. 640 *μ*g/g of total carotenoid with 355.9 *μ*g/g of *β*-carotene, 38 *μ*g/g of astaxanthin, 215 *μ*g/g of zeaxanthin, and 23 *μ*g/g of canthaxanthin. The spectrophotometric analysis revealed *β*-carotene and zeaxanthin as major carotenoids in all screened fungal strains.

Khanafari et al. [50] also investigated the carotene production potential of different *M. hiemalis* strains under different light incubations and reported 1.3 mg/g of total carotenoid under white light incubation. AUMC 6697.A was reported as the lowest producers of carotenoid (100 *μ*g/g) as its carotene content was found 6-fold less as compared to AUMC 6695, while in the current study, AUMC 6695 was found to produce 640 *μ*g/g of total carotenoid content, which might be attributed to its isolation from novel source and indicated by its bright orange color (textile polymer). Hence, it was concluded that strain AUMC 6695 can be further optimized for carotenoid production as a future scope of the study. Among *M. circinelloides* strains, AUMC 698 was found to be a higher producer of total carotenoid with 450 *μ*g/g of which *β*-carotene concentration was 250 *μ*g/g and zeaxanthin was produced in an amount of 165 *μ*g/g as a major carotenoid, while AUMC 11616.A was found as a low producer of total carotenoid (190 *μ*g/g) with 90 *μ*g/g of *β*-carotene and 81 *μ*g/g of zeaxanthin. Csernetics et al. [51] reported in their study that MS12 strain of *M. circinelloides* produced a total carotenoid content of 399 ± 27 of which *β*-carotene was a major carotene and produced in a concentration of 257 ± 18 *μ*g/g [51]. More recently, by Naz et al. [25], they reported that *M. circinelloides* strain CBSS 277.49 could produce up to 698.4 ± 3.68 *μ*g/g of *β*-carotene as a major carotene. Moreover, it was also concluded in the current study that *M. hiemalis* strains are more efficient for carotenoid production as compared to strains of *M. circinelloides*. It produced 1.42-fold more carotenoid as compared to *M. circinelloides* strains.

The results of the present experiment demonstrated that the fungal crude total lipids of all tested *Mucor* spp. induced a wider range of antimicrobial activity, due to the presence of high unsaturated fatty acids. The obtained results are in agreement with the findings of Ballantine et al. [52]; they found that some of the microbial lipid extracts exhibited antifungal and antibacterial activity against *Candida albicans*, *P. aeruginosa*, and *E. coli*. Ramadan et al. [53] showed that the lipid extract of *Spirulina platensis* induced antifungal activity against *A. niger* and *C. albicans* with an inhibition zone of 28.3 mm and 21.3 mm, respectively. In another study carried out by Patra et al. [54], they found that the lipid extract of *E. compressa* exhibited antibacterial activity against *B. subtilis* and *E. coli* with 14 mm and 12 mm inhibition zones, respectively. Therefore, this study confirmed that selected fungi have shown promising antimicrobial activity against different human pathogens which can help future drug discovery and their application. Medium and long-chain unsaturated FAs seem to be more active against Gram-positive than Gram-negative bacteria [55]. Differences in cell wall composition may allow better access to the bacterial membrane in Gram-positive compared to Gram-negative bacteria. [56].

## 5. Conclusions

In summary, 81 fungal isolates were isolated from the different sources, targeting the production of PUFA and carotenoids, where the fungus encoded as *R. pusillus* AUMC 11616.A was selected as a promising strain for total lipids and GLA. Also, *M. hiemalis* AUMC 6031 and *M. hiemalis* AUMC 6695 for carotenoid production. The evaluation of the total lipid of the fungus *R. pusillus* AUMC 11616.A exhibited satisfactory production of lipid and PUFA, mainly GLA. PUFAs are of great medical importance. Also, the high carotenoid content and consequently the antimicrobial activity of the total lipids indicate that these *R. pusillus* AUMC 11616.A and AUMC 6695 isolates have great industrial importance and can be processed into high value-added products, such as GLA.

## Figures and Tables

**Table 1:** Morphological identification of the selected fungal isolates and their isolation sources.

No.	Fungal code	Morphology	Fungal genus	Isolation source
1	AUMC 698	Mycelial	*Mucor*	Onion, Cairo
2	AUMC 697	Mycelial	*Mucor*	Soil, Alexandria
3	AUMC 6027	Mycelial	*Mucor*	Cow dung
4	AUMC 11616.A	Mycelial	*Mucor*	Cattle manure
5	AUMC 11641	Mycelial	*Mucor*	Soil, Assiut
6	AUMC 6031	Mycelial	*Mucor*	Horse dung
7	AUMC 6036	Mycelial	*Mucor*	Air of clover plant
8	AUMC 6695	Mycelial	*Mucor*	Textile 100% polyester
9	AUMC 6696.A	Mycelial	*Mucor*	Textile 100% polyester
10	AUMC 6697.A	Mycelial	*Mucor*	Textile polyester/cotton
11	AUMC 9172	Mycelial	*Mucor*	Case of laryngitis

**Table 2:** Biomass concentration (g/L), total lipids (g/L), total lipids per CDW (%), and glucose consumed of tested strains.

No.	Fungal strains	Biomass dry weigh (g/L)	Total lipid dry weight (g/L)	Total lipid percentage to biomass dry weight (%)	Lipid yield per gram of glucose (g/g)
1	AUMC 698	9.33	3.64	39.01	0.045
2	AUMC 697	9.35	3.48	37.21	0.043
3	AUMC 6027	6.38	2.25	35.26	0.028
4	AUMC 11616.A	7.73	4.00	51.74	0.050
5	AUMC 11641	9.44	3.80	40.25	0.047
6	AUMC 6031	9.38	3.66	39.01	0.045
7	AUMC 6036	9.38	3.48	37.10	0.043
8	AUMC 6695	9.61	3.75	39.02	0.046
9	AUMC 6696.A	9.15	4.00	43.71	0.050
10	AUMC 6697.A	10.44	2.27	21.74	0.028
11	AUMC 9172	8.90	3.33	37.41	0.041

**Table 3:** Fatty acid profile of 11 oleaginous strains.

Fungal strain	Composition of fatty acid (% TFA)						
	C14:0	C16:0	C16:1	C18:0	C18:1	C18:2	C18:3
AUMC 698	3.05 ± 0.11	22.95 ± 0.33	5.87 ± 0.14	3.15 ± 0.18	41.55 ± 1.25	13.85 ± 0.28	9.58 ± 0.06
AUMC 697	2.65 ± 0.18	23.01 ± 0.39	5.56 ± 0.40	3.61 ± 0.13	43.64 ± 1.25	13.66 ± 0.28	7.87 ± 0.08
AUMC 6027	15.93 ± 1.89	15.23 ± 0.08	12.43 ± 1.80	3.26 ± 0.33	27.61 ± 0.10	14.88 ± 0.70	10.65 ± 0.22
AUMC 11616.A	2.77 ± 0.11	20.47 ± 0.20	4.94 ± 0.10	3.46 ± 0.22	43.37 ± 1.77	14.40 ± 0.26	10.58 ± 0.81
AUMC 11641	2.59 ± 0.40	25.16 ± 0.89	5.07 ± 0.18	3.84 ± 0.65	41.20 ± 0.15	12.31 ± 0.52	9.83 ± 0.02
AUMC 6031	2.40 ± 0.40	23.91 ± 1.39	4.32 ± 0.66	3.67 ± 0.12	41.18 ± 0.15	14.09 ± 0.10	10.43 ± 0.22
AUMC 6036	2.84 ± 0.13	22.84 ± 0.49	5.09 ± 0.11	3.07 ± 0.08	42.13 ± 1.25	14.49 ± 0.10	9.54 ± 0.37
AUMC 6695	3.03 ± 0.18	24.11 ± 1.15	5.87 ± 0.34	6.31 ± 0.10	39.62 ± 0.81	11.78 ± 0.20	9.27 ± 0.58
AUMC 6696.A	2.61 ± 0.22	21.66 ± 0.42	4.88 ± 0.23	3.44 ± 0.01	44.02 ± 0.66	13.61 ± 0.81	9.78 ± 0.16
AUMC 6697.A	2.58 ± 0.11	21.69 ± 0.45	4.03 ± 0.74	4.46 ± 0.08	44.48 ± 1.21	14.49 ± 1.25	8.27 ± 0.11
AUMC 9172	2.67 ± 0.22	21.92 ± 0.08	4.84 ± 1.14	3.41 ± 0.06	43.30 ± 0.20	14.05 ± 1.21	9.81 ± 0.16

**Table 4:** Antimicrobial activity of extracted total lipids form tested strains against pathogenic microorganisms.

Fungal strain	Inhibition zone diameter (mm)					
	*S. aureus*	*B. subtilis*	*E. coli*	*S. typhi*	*A. flavus*	*P. chrysogenum*
AUMC 698	15 ± 0.17	13 ± 0.48	13 ± 0.15	14 ± 0.17	14 ± 0.12	15 ± 0.44
AUMC 697	14 ± 0.09	14 ± 0.07	12 ± 0.03	14 ± 0.16	15 ± 0.48	14 ± 0.18
AUMC 6027	12 ± 0.04	14 ± 0.20	13 ± 0.17	13 ± 0.03	14 ± 0.10	13 ± 0.10
AUMC 11616.A	14 ± 0.12	12 ± 0.20	12 ± 0.06	14 ± 0.20	14 ± 0.12	16 ± 0.17
AUMC 11641	15 ± 0.20	13 ± 0.48	13 ± 0.08	15 ± 0.48	15 ± 0.17	17 ± 0.12
AUMC 6031	15 ± 0.18	14 ± 0.34	13 ± 0.09	14 ± 0.34	12 ± 0.10	16 ± 0.20
AUMC 6036	16 ± 0.48	13 ± 0.26	12 ± 0.05	14 ± 0.20	14 ± 0.20	14 ± 0.32
AUMC 6695	14 ± 0.08	12 ± 0.05	13 ± 0.10	14 ± 0.41	15 ± 0.16	14 ± 0.16
AUMC 6696.A	14 ± 0.41	13 ± 0.10	14 ± 0.17	14 ± 0.41	13 ± 0.05	16 ± 0.12
AUMC 6697.A	14 ± 0.03	12 ± 0.06	15 ± 0.20	14 ± 0.17	13 ± 0.07	15 ± 0.17
AUMC 9172	13 ± 0.06	12 ± 0.17	13 ± 0.10	14 ± 0.06	11 ± 0.11	13 ± 0.30
Positive control	23 ± 0.02	23 ± 0.05	24 ± 0.03	23 ± 0.10	23 ± 0.10	23 ± 0.10
Negative control	No zone	No zone	No zone	No zone	No zone	No zone

**Table 5:** MIC values of extracted lipids from tested fungal strains against various pathogenic microorganisms (mg/mL).

Fungal strain	Tested human pathogens					
	*S. aureus*	*B. subtilis*	*E. coli*	*S. typhi*	*A. flavus*	*P. chrysogenum*
AUMC 698	100	50	50	75	50	50
AUMC 697	75	75	75	75	100	50
AUMC 6027	75	75	50	50	75	75
AUMC 11616.A	75	75	75	75	50	25
AUMC 11641	100	50	50	100	100	25
AUMC 6031	100	75	50	75	75	25
AUMC 6036	100	50	75	75	75	50
AUMC 6695	75	75	50	50	100	50
AUMC 6696.A	75	50	75	75	50	25
AUMC 6697.A	50	75	100	50	50	25
AUMC 9172	50	75	50	50	100	100

## Data Availability

No data were used to support this study.

## Supplementary Materials

Supplementary Materials Table S1: morphological and microscopic features of all fungal isolates in relation to their sources in this study.
