# Supplementary material for: Comparative Analysis of Different Isolated Oleaginous Mucoromycota Fungi for Their γ-Linolenic Acid and Carotenoid Production
Source: Biomed Res Int. 2020 Nov 5;2020:3621543. doi: 10.1155/2020/3621543 (PMC7665918; doi:10.1155/2020/3621543)
Supplement: Supplementary Materials — Table S1: morphological and microscopic features of all fungal isolates in relation to their sources in this study. [file 3621543.f1.docx]

**Table S1**. Morphological and microscopic features of fungal isolates in relation to their sources in this study

| Source | Number of isolates | Morphological identification | No. |
| --- | --- | --- | --- |
| Onion, Cairo | 1 | *Mucor spp.* | 1 |
|  | 3 | *Penicillium spp.* | 2 |
|  | 2 | *Aspergillus niger (group)* | 3 |
|  | 1 | *Aspergillus fumigatus* | 4 |
|  | 2 | *Fusarum spp.* | 5 |
| Soil, Alexanderia | 1 | *Mucor spp.* | 6 |
|  | 2 | *Aspergillus niger (group)* | 7 |
|  | 3 | *Penicillium spp.* | 8 |
|  | 2 | *Aspergillus versicolor* | 9 |
|  | 3 | *Trichoderma spp.* | 10 |
|  | 2 | *Alternaria spp.* | 11 |
|  | 1 | *Emercilla* | 12 |
|  | 2 | *Eppicoccum spp.* | 13 |
|  | 2 | *Aspergillus flavus* | 14 |
|  | 2 | *Cladosporium spp.* | 15 |
|  | 3 | *Fusarum spp.* | 16 |
| Cow dung | 1 | *Mucor spp.* | 17 |
|  | 3 | *Alternaria sp* | 18 |
|  | 1 | *Aspergillus fumigates* | 19 |
|  | 2 | *Penicillium spp.* | 20 |
|  | 1 | *Cladosporium spp.* | 21 |
|  | 1 | *Curvularia spp.* | 22 |
| Cattle manure | 1 | *Rhizomucor spp.* | 23 |
|  | 1 | *Aspergillus niger (group)* | 24 |
|  | 2 | *Yeasts* | 25 |
|  | 1 | *Aspergillus flavus* | 26 |
|  | 1 | *Phomatospora spp.* | 27 |
|  | 2 | *Podospora spp.* | 28 |
|  | 1 | *Geotrichum spp* | 29 |
| Soil, Assiut | 1 | *Mucor spp.* | 30 |
|  | 2 | *Trichoderma spp.* | 31 |
|  | 2 | *Aspergillus niger (group)* | 32 |
|  | 2 | *Alternaria spp* | 33 |
|  | 3 | *Fusarum spp.* | 34 |
|  | 2 | *Penicellium spp.* | 35 |
| Horse dung | 1 | *Mucor spp.* | 36 |
|  | 1 | *Podospora spp.* | 37 |
|  | 2 | *Aspergillus fumigates* | 38 |
|  | 2 | *Yeasts* | 39 |
|  | 2 | *Alternaria spp* | 40 |
| Air of clover plant | 1 | *Mucor spp.* | 41 |
|  | 1 | *Cladpsporium spp.* | 42 |
|  | 1 | *Aspergillus niger (group)* | 43 |
| Textile 100% polyester | 2 | *Mucor spp.* | 44 |
|  | 1 | *Yeasts* | 45 |
| Textile polyester/cotton | 1 | *Mucor spp.* | 46 |
| Case of laryngitis | 2 | Aspergillus spp | 47 |
|  | 1 | *Mucor spp.* | 48 |
